# Genomic analysis of primary plasma cell leukemia reveals complex structural alterations and high-risk mutational patterns

**DOI:** 10.1038/s41408-020-0336-z

**Published:** 2020-06-19

**Authors:** Carolina Schinke, Eileen M. Boyle, Cody Ashby, Yan Wang, Valeriy Lyzogubov, Christopher Wardell, Pingping Qu, Antje Hoering, Shayu Deshpande, Katie Ryan, Sharmilan Thanendrarajan, Meera Mohan, Naveen Yarlagadda, Maliha Khan, Samrat Roy Choudhury, Maurizio Zangari, Frits van Rhee, Faith Davies, Bart Barlogie, Gareth Morgan, Brian A. Walker

**Affiliations:** 1 Myeloma Center, Winthrop P. Rockefeller Cancer Institute, University of Arkansas for Medical Sciences, Little Rock, AR USA; 2 Cancer Research and Biostatistics, Seattle, WA USA; 3 Department of Hematology and Medical Oncology, Tisch Cancer Institute, Icahn School of Medicine at Mount Sinai, New York, NY USA; 4 Division of Hematology Oncology, School of Medicine, Indiana University, Indiana, IN USA

**Keywords:** Cancer genomics, Cancer genomics

## Abstract

Primary plasma cell leukemia (pPCL) is a rare and aggressive form of multiple myeloma (MM) that is characterized by the presence of ≥20% circulating plasma cells. Overall survival remains poor despite advances of anti-MM therapy. The disease biology as well as molecular mechanisms that distinguish pPCL from non-pPCL MM remain poorly understood and, given the rarity of the disease, are challenging to study. In an attempt to identify key biological mechanisms that result in the aggressive pPCL phenotype, we performed whole-exome sequencing and gene expression analysis in 23 and 41 patients with newly diagnosed pPCL, respectively. The results reveal an enrichment of complex structural changes and high-risk mutational patterns in pPCL that explain, at least in part, the aggressive nature of the disease. In particular, pPCL patients with traditional low-risk features such as translocation t(11;14) or hyperdiploidy accumulated adverse risk genetic events that could account for the poor outcome in this group. Furthermore, gene expression profiling showed upregulation of adverse risk modifiers in pPCL compared to non-pPCL MM, while adhesion molecules and extracellular matrix proteins became increasingly downregulated. In conclusion, this is one of the largest studies to dissect pPCL on a genomic and molecular level.

## Introduction

Primary plasma cell leukemia (pPCL) is a rare form of multiple myeloma (MM) defined by the presence of ≥20% circulating plasma cells (PCs) and/or an absolute PC count of 2 × 10^9^/L at diagnosis^1,2^. It is distinguished from secondary plasma cell leukemia (sPCL) by presenting at MM diagnosis, whereas sPCL is a leukemic transformation of end-stage MM. pPCL is characterized by an aggressive course with poor outcome and short overall survival (OS) of around 24 months^3–6^. Despite the advances of anti-MM therapy in recent years, patients with pPCL continue to have dismal survival suggesting that the underlying biology in pPCL differs substantially from patients with non-pPCL MM^3,7^. Mechanisms that underlie pPCL pathophysiology and distinguish it from MM remain incompletely understood despite being crucial to improve management. Previous reports have shown pPCL to be a highly complex disease enriched in adverse risk genetic aberrations, in particular a higher prevalence of deletions 13q and 17p, amplification of 1q as well as translocation t(14;16) compared to MM^7–12^. While these findings show that pPCL shares biological features of high-risk MM that partially explain the poor risk outcome, some pPCL patients present with traditional low-risk features such as the t(11;14) or hyperdiploidy, and yet will still have a poor outcome. Furthermore, the ability of pPCL cells to become independent of the surrounding bone marrow (BM) niche, which is shown to be crucial for non-pPCL MM to promote tumor growth and disease progression, suggests a distinct disease biology. Identifying mechanisms that underlie pPCL pathophysiology and distinguish it from MM remain incompletely understood and will be crucial to explore therapeutic options. Given the rarity of pPCL with only 1–2% of MM patients being affected, comprehensive studies remain challenging and have included small patient numbers. To address this lack of knowledge, we performed whole-exome sequencing (WES) and gene expression profiling (GEP) on 23 and 41 newly diagnosed pPCL patients, respectively, that presented to the Myeloma Center, University of Arkansas for Medical Sciences, Little Rock, USA from 2003 to 2018. This is one of the largest studies to date to effectively characterize the molecular spectrum of pPCL and to identify new avenues for therapeutic options in this poor risk disease.

## Methods

### Patients and samples

Specimens were obtained after approval from the institutional review board of the University of Arkansas for Medical Sciences in accordance with the Declaration of Helsinki. For WES, we included a total of 23 patients and for GEP, a total of 41 newly diagnosed pPCL patients that presented to the Myeloma Center, UAMS from 2003 to 2018. CD138+ plasma cells were isolated from BM aspirates by magnetic-activated cell sorting using the AutoMACS Pro (Miltenyi Biotec GmbH, Bergisch Gladbach, Germany) or RoboSep (STEMCELL Technologies, Vancouver, Canada). Plasma cell purity was determined by flow cytometry and only samples with >85% purity were used in this study. For matched nontumor control for each patient to exclude germline variants, we used CD34+ cells from stem cell harvest (*n* = 20) or cells obtained from buccal swabs (*n* = 3). Nucleic acids were isolated using the AllPrep DNA/RNA or Puregene kits (Qiagen, Hilden, Germany).

### Sequencing

Exome sequencing and mutation calling DNA from tumor samples were used in the exome capture protocol as previously published^13^ and sequenced on a NextSeq500 (Illumina) using 76-bp paired-end reads. FastQC (v0.10.0) was used for basic quality control of Illumina paired-end sequencing data. Exome alignment was performed to human reference genome hg38 using BWA (v.0.7.17) and duplicates marked using Sambamba (v.0.6.3). Somatic mutations were called using Strelka (v.2.8.3) with default parameters and exome flag set, filtered using fpfilter and annotated using Variant Effect Predictor (v.85). Structural variants were called using Manta (v.1.1.1) with default parameters and exome flag set. Copy number variants were called using Control-FREEC (v.11.0). The mean coverage was 90× (range: 57–326). Data have been deposited at dbGAP under accession number phs002022.v1.p1. Extraction of the APOBEC mutational signature was performed using the NNMF algorithm across cumulative catalogs of coding and noncoding mutations as previously described^14,15^.

### External datasets

This pPCL dataset was compared to previously published MGUS^16^ and MM^17,18^ datasets, which are available under accession numbers EGAS00001001658, EGAS00001001147, and EGAS00001000036. In brief, tumor load of nonsynonymous mutations was compared between pPCL, MM, and MGUS and copy numbers as well as prevalence of previously identified driver mutations were compared between pPCL and MM. Data were filtered similarly to ensure comparability of mutation numbers and the datasets were comparable in terms of sequencing technique, filtering, and depth as shown in Supplementary Table 1.

### Gene expression profiling

Gene expression profiling analysis of total RNA from plasma cells was performed with the Affymetrix U133 Plus 2.0 microarray platform (Santa Clara, CA, USA) using methods previously described^19,20^. GEP was available from 41 newly diagnosed pPCL patients, 15 of which had paired GEP from plasma cells of the peripheral blood and BM aspirate. Gene expression profiling of pPCL patients (*n* = 41) was compared to 739 newly diagnosed MM patients, 42 patients with monoclonal gammopathy of undetermined significance (MGUS), 73 patients with smoldering MM (SMM) and 34 healthy donors (HD). Samples used for gene expression analysis were derived from previous trials and have been published previously^3,19,21,22^.

### Statistical analysis

Differences between copy number alterations and prevalence of driver mutations between pPCL and MM were analyzed using the chi-square test. Significant alterations in mutational load of nonsynonymous mutations between pPCL, MM, and MGUS were measured using the Student’s *t* test. Hierarchical clustering of paired peripheral blood and BM samples was performed using the Euclidean distance method with average linkage. Differential GEPs were analyzed comparing the log expression of samples from pPCL and samples from non-PCL MM patients using the limma procedure^23^. Genes with a criterion of False Discovery Rate (FDR) < 0.01 and fold change ≥ 2 were selected, leading to 24 upregulated and 102 downregulated genes in pPCL. The volcano plot of mean log expression difference between the two groups in the *x-*axis versus −log10 (FDR) in the *y*-axis was created with R (version 3.4). Unsupervised hierarchical clustering of paired pPCL cells from peripheral blood and BM aspirate was performed using the hclust function in R (version 3.4).

## Results

### Patients’ characteristics

Whole-exome sequencing was performed on 23 newly diagnosed pPCL patients, and clinical baseline characteristics were compared to a cohort of previously published MM patients^17,18^, wherever data were available. The vast majority of patients presented with adverse clinical markers including ISS III, elevated LDH as well as renal impairment (Table 1). Significantly more patients with pPCL presented with ISS stage III compared to MM patients and had adverse genetic risk features such as significantly higher proportion of maf translocations [t(14;16) and t(14;20)], increased proportion of chromosome 1q gain, and fewer hyperdiploid patients. Maf-subgroup translocations were highly enriched in this pPCL cohort with 35% having a t(14;16) involving *MAF* and 9% a t(14;20) involving *MAFB*. Up to a third of patients presented with standard risk primary genetic events, including hyperdiploidy, defined by the gain of at least two odd chromosomes, and t(11;14)^24^. Yet OS between cytogenetic subgroups in the pPCL group did not differ significantly and was overall poor (median OS = 23 months), suggesting that other molecular events overcome initial low-risk features and impact prognosis. The prevalence of adverse risk *MYC* alterations in pPCL was similar to MM^25^. This includes *MYC* translocations to immunoglobulin heavy- and light-chain loci and rearrangements with known or expected superenhancers^25^ on chromosomes 1p12 (FAM46C), 3q26 (MYNN), 5q33 (SPARC), 6q21 (QRSL1), 7q32 (PLXNA4), 15, 18q22 (CDH7) in 35% of all pPCL patients, as well as *MYC* gain, inversion, and deletion in 13%, 17%, and 4% of all pPCL samples, respectively.

**Table 1:** Patient Characteristics of the pPCL cohort compared to a previously published MM cohort^17^^,18^.

Patient clinical characteristics	pPCL (*n* = 23)	MM (*n* = 1273)	*p* value
Median age at diagnosis (Min−Max)	59 (36–77)	66 (27–93)	
Age ≥ 65 years (%)	9/23 (39%)	693/1273 (54%)	NS
Isotype (Kappa/Lambda)	11/12	—	
ISS stage I (%)	0/23 (0%)	360/1170 (31%)	<0.01
ISS stage II (%)	1/23 (4%)	442/1170 (38%)	<0.01
ISS stage III (%)	22/23 (96%)	368/1170 (31%)	<0.0001
Elevated LDH (%)	13/17 (76%)	—	
Elevated creatinine (≥2 mg/dL)	18/22 (81%)	—	
t(11;14)	5/23 (22%)	234/1273 (18%)	NS
t(14;16) or t(14;20)	10/23 (43%)	62/1273 (5%)	<0.0001
t(4;14)	2/23 (9%)	58/461 (13%)	NS
Hyperdiploidy	3/23 (13%)	456/1074 (42%)	<0.01
Gain of chromosome 1q	17/23 (74%)	314/1074 (29%)	<0.0001
1q duplication (=3 copies)	13/23 (57%)	—	
1q amplification (≥4 copies)	4/23 (17%)	—	
C-Myc translocations			
Ig partner loci (IGH, IGK, IGL)	5/23 (22%)	202/751 (27%)	NS
Non-Ig partner loci (FAM46C, MYNN, SPARC, QRSL1, PLXNA4, CDH7, RNF123)	9/23 (39%)	—	

### Copy number alterations

**Fig. 1:**
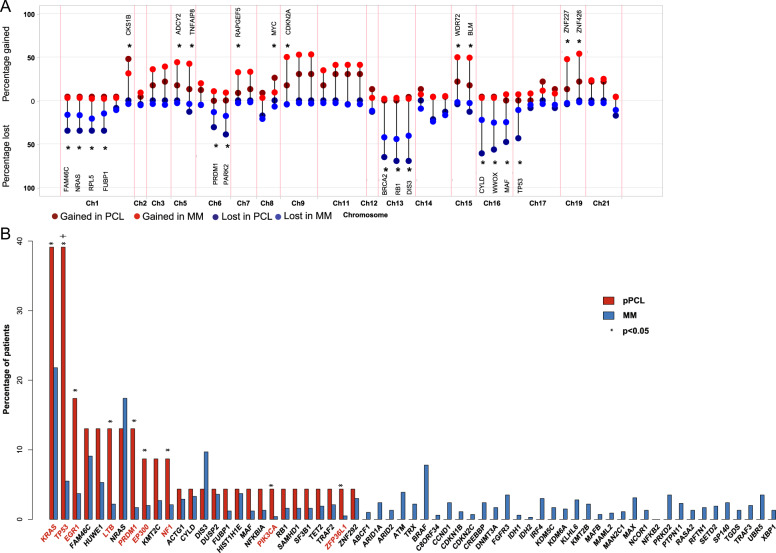
The prevalence of copy number alterations and driver mutations in pPCL compared to MM. **a** pPCL is enriched for adverse risk copy number alterations compared to MM^17^, *p < 0.05. Significant genes are labeled, with the exception of markers on chromosomes associated with hyperdiploidy. **b** The frequency of driver mutations in pPCL and MM, *p < 0.05 (chi-square test); ^+^*p* < 0.005 (after multiple test correction).

Copy number alterations (CNA) were consistent with a high-risk signature and are shown in Fig. 1a and Supplementary Table 2. The plot shows how the gain/loss of significant driver genes that are representative of their chromosome segment differ in pPCL to previously published MM data^17,18^. Copy number variations of whole chromosome segments in pPCL are shown in Supplementary Fig. 1. When compared to MM patients, the whole cohort of pPCL showed significantly more cases with deletion of 1p, 6q, 13q, 16q, and 17p as well as a significant gain of chromosome 1q compared to MM, all of which have previously shown to convey poor prognosis^26,27^. Hyperdiploidy^28^ was less prevalent compared to MM, consistent with previous reports^8,12,29^. Biallelic inactivation of *TP53* was seen in 35% (8/23) of patients (Supplementary Table 3) and was particularly enriched in t(11;14) patients (60%), offering a possible explanation for the adverse outcome observed in this traditionally low-risk primary MM subtype.

To investigate whether these CNA are merely attributable to differences in hyperdiploid cases between pPCL and MM, we further analyzed the CNA between nonhyperdiploid (nHRD) pPCL samples (*n* = 20) compared to their nHRD MM counterpart (*n* = 456) and still saw significant differences in deletion of 1p, 6q, 16q, and *TP53* (Supplementary Fig. 2, Supplementary Table 4). While more cases of nHRD pPCL showed a gain of 1q compared to nHRD MM, the result was nonsignificant. Of interest, genes on chromosome 8q (*MYC*), 9q (RNF20, TRAF2), 15q (WDR72, BLM), and 21q (CHODL, SON) demonstrated a significant gain in nHRD pPCL patients compared to nHRD MM patients, which was not observed when comparing the whole pPCL and MM cohort to each other.

### Driver mutations

Further, we determined the prevalence of previously identified driver mutations that have been shown to promote tumor growth and predict worse prognosis in MM^17,30^. Chi-square analysis showed that several driver genes (*KRAS*, *TP53*, *EGR1*, *LTB*, *PRDM1*, *EP300*, *NF1*, *PIK3CA*, and *ZFP36L1)* had significantly more mutations in pPCL compared to MM (*p* < 0.05). However, after adjusting for sample size and multiple test correction, only *TP53* was mutated at a significantly higher frequency in pPCL compared to MM (*p* < 0.005, Fig. 1b). Mutational sites of *KRAS* and *TP53*, the most frequently mutated genes, are shown in Supplementary Table 5 and show a pattern similar to MM. Of note is that mutations in *BRAF*, which increase MAPK activation similar to *KRAS* and *NRAS*, were absent in this patient cohort. Compared to non-pPCL MM, Ras mutations, including *KRAS* and *NRAS*, were particularly common in the t(14;16), t(14;20), t(4;14), and hyperdiploid subgroups, while *TP53* mutations were highest in the t(11;14) (Supplementary Fig. 3). Similar to alterations in copy number variations, we additionally compared the spectrum of driver mutations in nHRD pPCL to nHRD MM (Supplementary Fig. 4). Mutations of *TP53* remained significantly higher in the nHRD pPCL cohort.

### Mutational burden

**Fig. 2:**
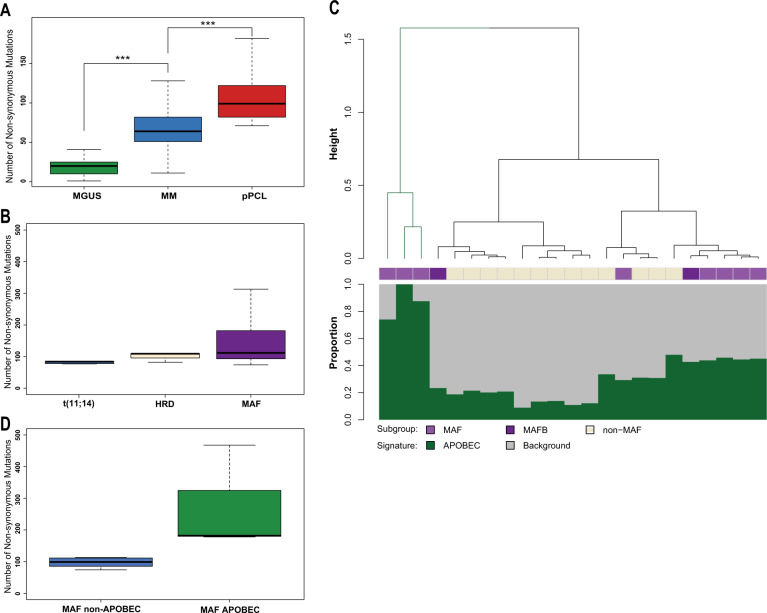
Mutational burden and the prevalence of the APOBEC signature in pPCL. **a** The median burden of nonsynonymous mutations increases with disease stage. pPCL (median 99, range 74–468), MGUS (median 20, range 1–66), and MM (median 64, range 11–2263), ***p < 0.001. **b** The prevalence of the APOBEC signature in the pPCL cohort. Only three patients (13% of total group and 33% of patients with MAF translocations) had a high percentage of the APOBEC signature, which is lower than reported in non-pPCL MM. **c** The amount of nonsynonymous mutations per cytogenetic subgroup is shown in (**a**). The median number of nonsynonymous mutations is relatively high in each subgroup: t(11;14) (median = 84, mean = 84), hyperdiploidy (HRD) (median = 104, mean = 100) and MAF including t(14;16) and t(14;20) (median = 112, mean = 171). There was no statistical significant difference between these groups. **d** The mutational load within the maf-subgroup between patients with a high APOBEC contribution compared to those with a not substantially increased APOBEC contribution.

The number of nonsynonymous mutations per sample was increased in the pPCL group (median = 99) and was significantly higher compared to other subtypes (MGUS median, *n* = 20, MM median, *n* = 64)^16,17^ (Fig. 2a). The high number of nonsynonymous mutations in the pPCL cohort was not solely due to the high percentage of samples with maf translocations in this cohort, which are known to have an elevated number of mutations in MM patients due to an APOBEC mutational signature^31,32^. The median number of nonsynonymous mutations in the t(11;14), hyperdiploid, and t(14;16) pPCL samples was 84, 109, and 112, respectively (Fig. 2b). There was no significant difference in the number of nonsynonymous mutations between these groups. However, the maf patients had a wide range of nonsynonymous mutations per sample. Mutational signature analysis of the pPCL samples indicated that the minority of pPCL samples had the APOBEC signature, including patients with maf translocations, where only 3/10 (30%) had a significant proportion of APOBEC mutations (Fig. 2c). This finding contrasts dramatically from the analysis of MM cohorts where approximately 80% of patients with maf translocations demonstrated an APOBEC mutational signature, which was associated with poor outcome^32^. Interestingly, when splitting maf-group pPCL patients by the presence of APOBEC mutational signature, the median number of nonsynonymous mutations in the APOBEC maf-group is still significantly higher compared to those without the APOBEC signature (182 and in the non-APOBEC maf-group is only 96, Fig. 2d), indicating that APOBEC still contributes to genomic instability in APOBEC-positive cases. Yet, there was no difference in outcome between patients with or without the APOBEC signature (data not shown) and the low incidence of APOBEC in this enriched maf-positive cohort suggests that the APOBEC-derived signature is not a primary driver of pPCL disease.

### Gene expression analysis

**Fig. 3:**
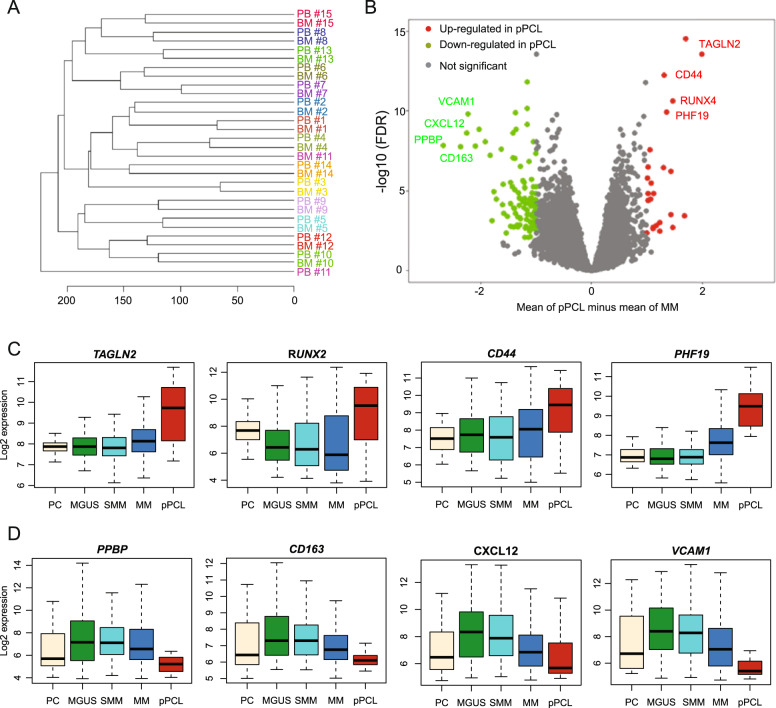
Gene expression differences in pPCL. **a** Unsupervised clustering of matched CD138+ cells from BM aspirates and PB from the same patients (*n* = 15). **b** Gene expression profiling comparing differentially expressed genes between non-pPCL MM and pPCL with at least a twofold change (*p* < 0.05, FDR < 0.01). **c** Expression of the top four upregulated genes by MM stage and in healthy donors (HD). **d** The top four downregulated genes by MM stage and in HD.

To gain additional insights into the underlying mechanisms, we performed GEP analysis of BM aspirates in 41 newly diagnosed pPCL patients, of which 15 patients had paired CD138+ cells from peripheral blood (PB). Unsupervised clustering showed that in 93% of cases, pPCL cells derived from BM and PB clustered together (Fig. 3a), underscoring previous findings that circulating pPCL cells are genetically and phenotypically similar to BM-derived pPCL cells^8^. To identify transcriptional changes that are pertinent to pPCL, we compared the gene expression signature of the 41 pPCL patients to the gene expression profile of 739 newly diagnosed non-pPCL MM patients. Significant differentially expressed gene probes with at least twofold change are shown in Supplementary Table 6. Interestingly, of these 126 significantly differentially expressed genes, the majority were downregulated in pPCL compared to non-pPCL MM (Fig. 3b). None of these differentially expressed genes was significantly mutated or had altered copy number, suggesting a different mechanism of expression regulation. The top four up- and downregulated genes between pPCL and non-pPCL MM were plotted according to their disease stage (Fig. 3c, d, healthy donor [*n* = 34], MGUS [*n* = 42], SMM [*n* = 73], MM [*n* = 739], pPCL [*n* = 41]). The top four upregulated genes display a pattern of continuous increase of expression that correlated with disease aggressiveness, suggesting that these genes might play an important role in MM biology. In fact, *TAGLN2*^33^, *RUNX2*^34^, *CD44*^35,36^, and *PHF19*^37,38^ have all been shown to contribute significantly to MM pathophysiology or have prognostic implication. Most of the significantly downregulated genes were adhesion molecules present in the extracellular matrix, giving one possible explanation of how pPCL cells become detached and independent of the surrounding BM microenvironment. Interestingly, downregulation of adhesion molecules has previously shown to have adverse prognostic effects in pPCL^39^.

## Discussion

Whole-exome sequencing and GEP have greatly broadened our knowledge of molecular alterations leading to transformation and progression in MM; however, the molecular characterization of pPCL remains challenging, largely due to the rarity of the disease and small patient sizes. Cifola et al.^11^ previously reported on WES in a series of 12 pPCL patients and showed that pPCL is a highly complex and heterogenous disease with an increased mutational burden compared to MGUS/SMM and non-pPCL MM. An increase of nonsynonymous mutations, which has been shown to predict for worse survival and genome instability, was also seen in our dataset along with other adverse risk factors such as a high prevalence of *TP53* mutations as well as biallelic inactivation of *TP53*, which in our analysis were particularly evident in the t(11;14) subgroup. Genetic events that lead to biallelic inactivation of tumor suppressor genes have been strongly associated with early relapse in non-pPCL MM, indicating that these events lead to downstream mechanisms that enhance the evolutionary fitness and outgrowth of other clones^40,41^. Our data indicate that biallelic inactivation of *TP53* occurs early on in the oncogenesis of pPCL and likely plays a major role in therapy resistance and aggressive disease course.

When looking at the mutational spectrum, we observed some differences between our pPCL dataset and the previously reported one by Cifola et al.^11^. For example, *KRAS* mutations were highly prevalent in our pPCL cohort while not seen in the previous study. In contrast, *DIS3* mutations were seen in 25% of samples in the previous study, but were not common in our dataset. The reason for these discrepancies could be the comparison between two relatively small patient cohorts with different cytogenetic composition. For example, *DIS3* mutations have been shown to be prevalent in t(4;14)^17^, but this cytogenetic subgroup was relatively small in our pPCL cohort. Hyperdiploidy, which has been associated with an increased amount of *KRAS* mutations in MM^42^, was seen in three patients in our pPCL cohort, all of which had a *KRAS* mutation. In contrast, hyperdiploid cases have been described as absent or not prevalent in previous pPCL studies^7,11,12^, giving one possible explanation for the higher amount of *KRAS* mutations in our cohort. Furthermore, *KRAS* mutations were highly prevalent within the maf-subgroup, and this association has not been commonly observed in non-pPCL MM^17,30,43^. The absence of *BRAF* mutations in our pPCL cohort is somewhat surprising, particularly as it has been associated with t(14;16)^17^ and poor prognosis^44^. However, a low prevalence of *BRAF* mutations in pPCL has been described previously by Cifola et al.^11^ and Mosca et al.^9^, suggesting that this mutation might not be of high importance in pPCL.

It is also of interest that the APOBEC signature was not very prevalent in our cohort; only 13% of all patients showed a high APOBEC contribution. This observation was unexpected, in particular given the high mutational load of pPCL and a high prevalence of the maf-subgroup, which both have been associated with the APOBEC signature. Yet others similarly reported a substantial variation of the APOBEC signature in a small cohort of pPCL patient and human MM cell lines, with some samples having a high and others a rather low APOBEC contribution^45^. These results indicate a reduced involvement of the APOBEC-driven mutational signature in pPCL, suggesting alternative mechanisms of disease progression in pPCL further underscoring the genomic differences between MM and pPCL.

Furthermore, gene expression analysis revealed that genes that have been shown to be relevant in myeloma tumorigenicity become significantly upregulated in pPCL compared to non-pPCL MM. Of particular interest is the overexpression of *PHF19*, an epigenetic regulator, which has recently been shown to be a strong prognostic factor and a promising therapeutic target^34,36–38^. Additionally, the alteration in expression of adhesion molecules and extracellular matrix proteins is common to all molecular studies published on pPCL to date and underscores the importance of these molecules in the pathogenesis of pPCL^11,39^. Taken together our study adds valuable insight into the molecular landscape of pPCL and shows that pPCL is enriched for adverse risk genetic events, yet is still characterized by significant heterogeneity. While there are no distinct genetic features that distinguish pPCL from non-pPCL MM, pPCL is characterized by the accumulation of high-risk genetic events that lead to a distinct clinical picture evident by therapy resistance and poor overall prognosis. Elucidated pathways and candidate genes demonstrated in this work could be useful for novel future therapeutic approaches.

## Supplementary information

Supplemental File

## Data Availability

Whole-exome sequencing data are available at dbGAP under accession number phs002022.v1.p1.
