# Supplementary material for: Genomic analysis of primary plasma cell leukemia reveals complex structural alterations and high-risk mutational patterns
Source: Blood Cancer J. 2020 Jun 19;10(6):70. doi: 10.1038/s41408-020-0336-z (PMC7303180; doi:10.1038/s41408-020-0336-z)
Supplement: Supplementary file 1 — Supplemental File [file 41408_2020_336_MOESM1_ESM.pdf]

Genomic Analysis of Primary Plasma Cell Leukemia reveals complex Structural Alterations and High Risk Mutational Patterns

Supplementary Material

Authors: Carolina Schinke[1], Eileen M. Boyle[1], Cody Ashby[1], Yan Wang[1], Valeriy Lyzogubov[1], Christopher Wardell[1], Pingping Qu[2], Shayu Deshpande[1], Katie Ryan[1], Sharmilan Thanendrarajan[1], Meera Mohan[1], Naveen Yarlagadda[1], Maliha Khan[1], Samrat Roy Choudhury[1], Maurizio Zangari[1], Frits van Rhee[1], Faith Davies[1], Bart Barlogie[3], Gareth Morgan[1] and Brian A. Walker[1,4]

Affiliations:

1. Myeloma Center, Winthrop P. Rockefeller Cancer Institute, University of Arkansas for Medical Sciences, Little Rock, AR, USA.
2. Cancer Research and Biostatistics, Seattle, WA
3. Department of Hematology and Medical Oncology, Tisch Cancer Institute, Icahn School of Medicine at Mount Sinai, New York, NY
4. Division of Hematology Oncology, School of Medicine, Indiana University, Indiana, IN

Corresponding Author: Carolina Schinke and Brian Walker

Carolina Schinke, Myeloma Center, University of Arkansas for Medical Sciences, W Markham St, Little Rock, AR 72205. email: cdschinke@uams.edu

Brian Walker, Division of Hematology Oncology, Indiana University, IB 544B, 975 W Walnut St, Indianapolis, IN 46202. email: bw75@iu.edu

Supplemental Figure 1. Copy number alterations shown as loss and gain of whole chromosome segments in pPCL (n=23).

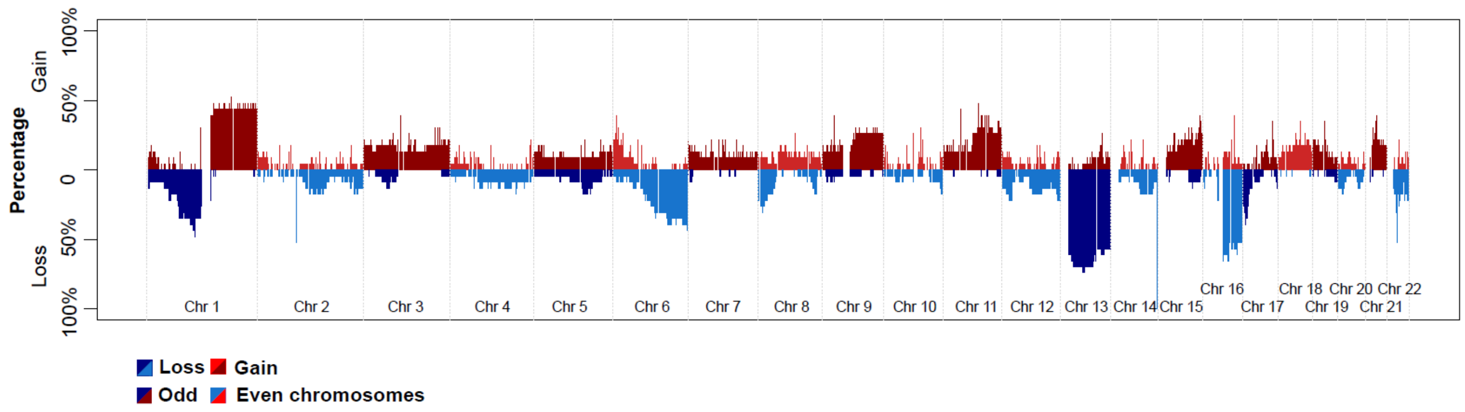

Supplemental Figure 2. Copy number alterations in non-hyperdiploid (nHRD) pPCL (n=20) and nHRD MM (n=456) cases.

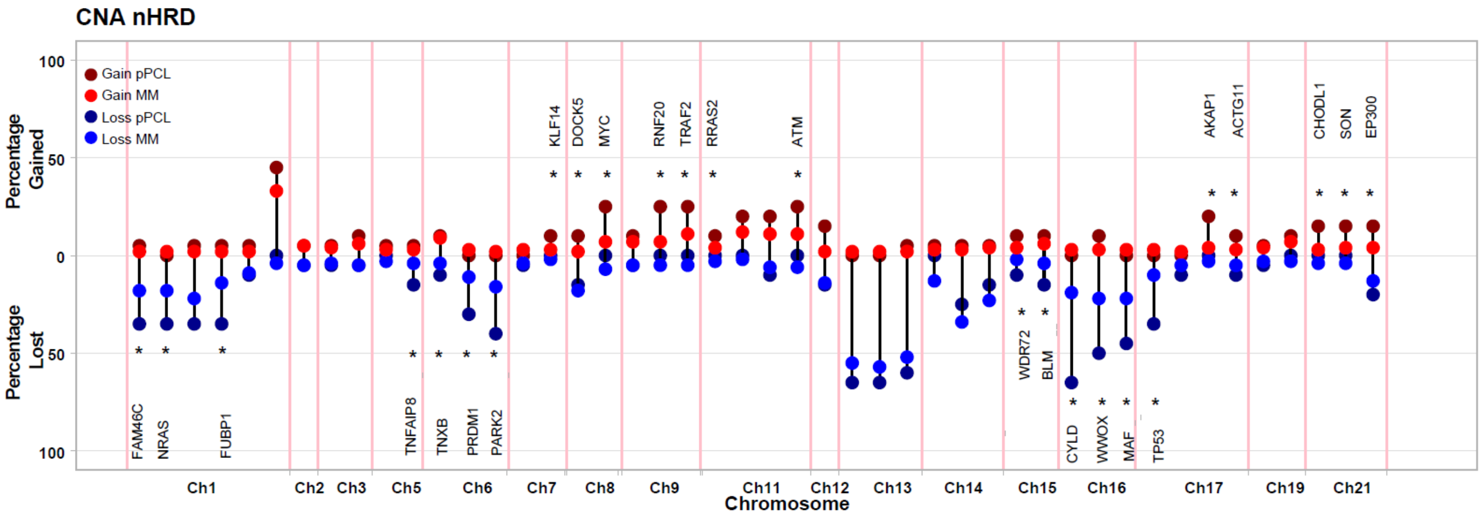

Supplemental Figure 3. The prevalence of *KRAS*, *NRAS* and *TP53* mutations per cytogenetic subgroups within this pPCL cohort compared to a previously published MM cohort. Total number of patients within each subgroup were annotated in **Table 1**. Though there are apparent substantial differences between pPCL and MM, these were non-significant given the small sample numbers and after adjusting for sample size.

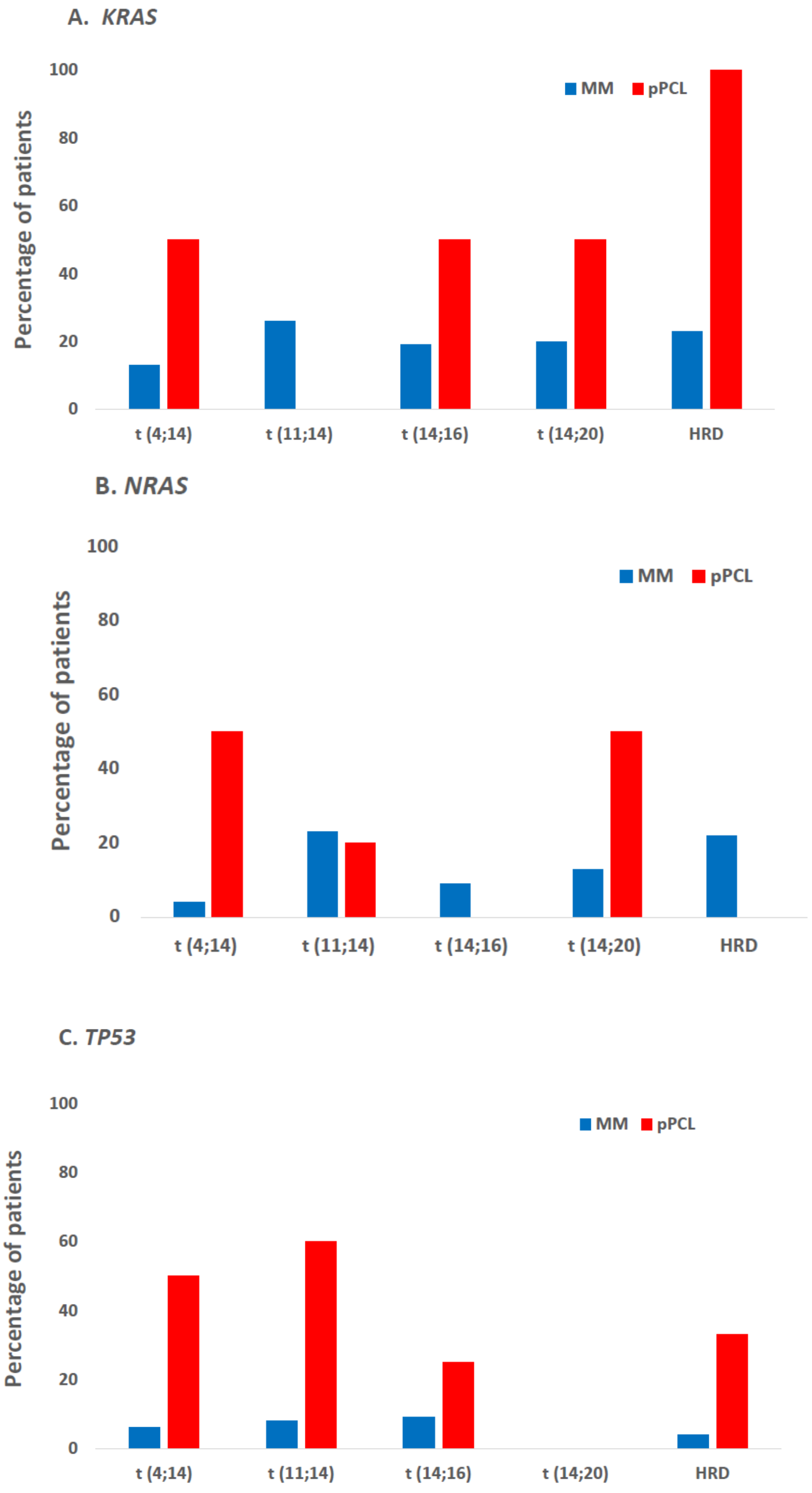

Supplemental Figure 4. The prevalence of driver mutations in non-hyperdiploid (nHRD) pPCL (n=20) and nHRD MM (n=456) cases, *= p<0.05.

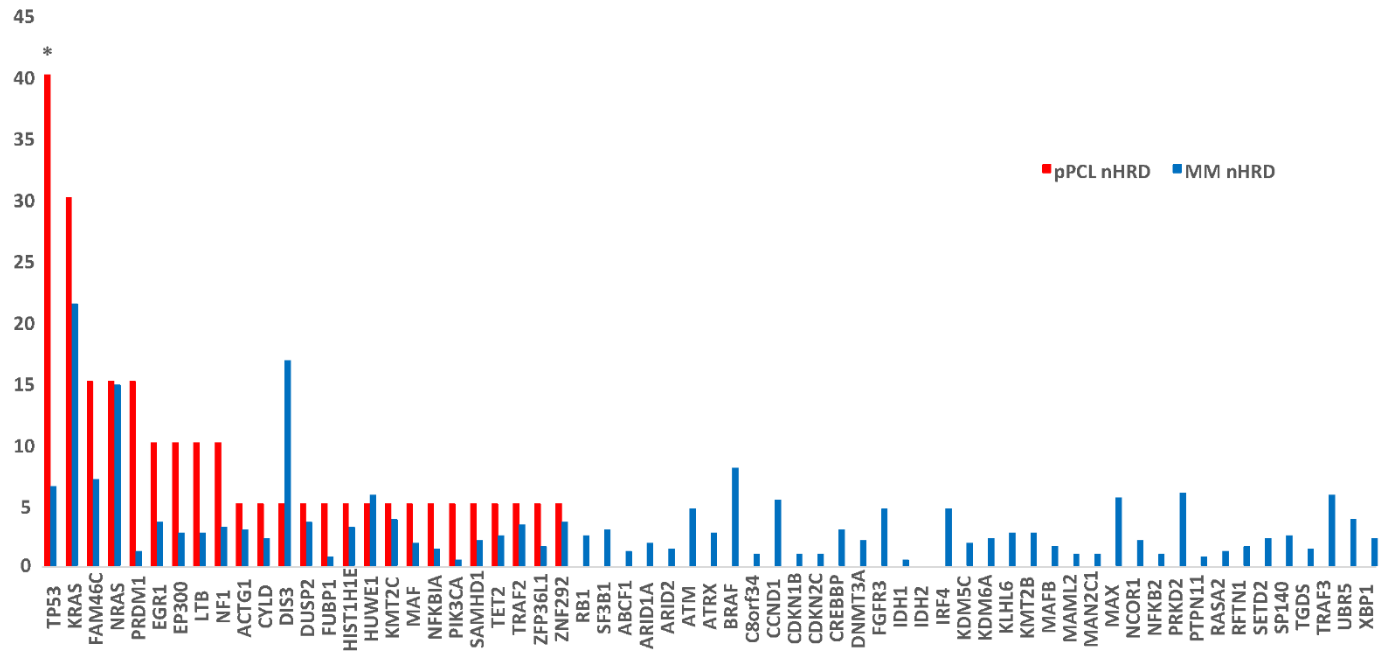

Supplemental Table 1. Similar Sequencing Methods from different datasets. This pPCL dataset was compared to previously published MGUS[1] and MM[2,3] datasets, which are available under accession numbers EGAS00001001658, EGAS00001001147 and EGAS00001000036. In brief, tumor load of nonsynonymous mutations was compared between pPCL, MM and MGUS and copy numbers as well as prevalence of previously identified driver mutations were compared between pPCL and MM. Data were filtered similarly to ensure comparability of mutation numbers. The datasets were comparable in terms of sequencing technique, filtering and depth. Of note, the MGUS samples had been sorted using flow then amplified using repli-G. This approach enables better purity and does not create any additional mutations, as shown by Mikulasova et al. Copy number was no longer evaluable therefore requiring additional copy number arrays.

	pPCL	MM (MGP)	MGUS
Mean depth	90x	107x	67x
Purity	>90%	>90%	Median:99%
Amplification	No	No	REPI-g Midi
Reads	76-bp paired-ends reads	76-bp paired-ends reads	76-bp paired-ends reads
Platform	NextSeq500	NextSeq 500	HiSeq 2000
Newly diagnosed	Yes	Yes	Yes
Variant calling	Strelka	MuTect/Strelka	MuTect

Supplemental Table 2. Copy number variations (CNV) between pPCL and MM. For this analysis, we looked at MM driver genes as previously defined and determined their CNV. Driver genes, whose copy number was altered in at least 2 samples are listed and percentages were compared to a previously published multiple myeloma (MM) dataset[2] using the Chi-Square test.

Gene	CHROMOSOME	pPCL Loss %	pPCL Gain %	MM Loss %	MM Gain %	p-value
FAM46C	1p12	34.8	4.3	14.5	1	0.007
NRAS	1p13.2	34.8	4.3	15	1	0.009
RPL5	1p22.1	34.8	4.3	18.9	0	0.01
FUBP1	1p31.1	34.8	4.3	13	0	0.002
CDKN2C	1p32.3	8.7	4.3	9	1	0.8
CKS1B	1q21.3	0	47.8	2	29.2	0.04
DNMT3A	2p23.3	4.3	4.3	3.4	7	0.65
CRBN	3p26.2	0	17.4	2	33.9	0.1
PIK3CA	3q26.32	0	21.7	3	37	0.1
ADCY2	5p15.3	0	17.4	1	42	0.02
TNFAIP8	5q23.1	13	13	2	40.3	0.01
TNXB	6p21.3	4.3	13	2	18.7	0.13
PRDM1	6q21	30.4	0	11	9	0.004
PARK2	6q26	39.1	0	15.8	7	0.002
RAPGEF5	7p15.3	0	8.7	1	30.5	0.02
KLF14	7q32.2	0	13	0	31	0.06
DOCK5	8p21.2	17.4	8.7	19.2	1	0.7
MYC	8q24.2	0	26.1	5	7.3	0.002
CDKN2A	9p21.3	4.3	17.4	2.6	48	0.004
RNF20	9q31.1	0	30.4	1	50.7	0.1
TRAF2	9q34.3	0	30.4	1.5	51	0.1
RRAS2	11p15.2	0	17.4	1	32.7	0.1
CCND1	11q13.3	0	30.4	1	38.9	0.11
BIRC3	11q22.1	4.3	30.4	2.7	39	0.8
ATM	11q22.3	0	30.4	2.3	39	0.8
CDKN1B	12p13.1	13	13	9.9	1	0.9
BRCA2	13q13.1	65.2	0	40.4	0	0.02
RB1	13q14.2	69.6	0	42.4	1	0.001
DIS3	13q21.33	69.6	4	38.6	0	<0.001
FGFR3	14p16.3	0	13	7.4	5	0.6
ABCD4	14q24.3	21.7	4.3	22.3	2	0.9
TRAF3	14q32.32	13	4.3	14.9	3	0.6
WDR72	15q21.3	4.3	21.7	0	47.7	0.01
BLM	15q26.1	13	17.4	1	47.2	0.005
CYLD	16q12.1	60.9	4.3	20.4	1	<0.001
WWOX	16q23.1	56.5	4.3	23.5	1	<0.001
MAF	16q23.2	47.8	0	23	5	0.005
TP53	17p13.1	43.5	0	9	5	<0.001
NF1	17q11.2	8.7	0	3	6	0.26
AKAP1	17q22	0	21.7	2	9.3	0.05

ACTG1	17q25.3	8.7	13	3	6	0.26
ZNF227	19q13.3	4.3	13	1	45.5	0.001
ZNF426	19p13.2	0	21.7	0	51.8	0.004
CHODL	21q21.1	0	21.7	1	21.2	0.9
SON	21q22.1	0	21.7	1	22.8	0.86
EP300	22q13.2	17.4	4.3	9	2	0.14

Supplemental Table 3. Incidence of biallelic inactivation of known tumor suppressor genes

Gene	pPCL (n=19)	MM (n=1074)*	p- value
DIS3	2% (1/23)	6.4% (69/1074)	NS
FAM46C	2% (1/23)	4.2% (45/1074)	NS
RB1	2% (1/23)	1.7% (18/1074)	NS
TP53	35% (8/23)	3.7% (40/1074)	<0.00001

Supplemental Table 4. Copy number variations (CNV) between non-hyperdiploid (nHRD) pPCL (n=20) and nHRD MM (n=456). Alterations of copy number variations of driver genes are listed and percentages were compared to a previously published multiple myeloma (MM) dataset[2] using the Chi-Square test.

Gene	CHROMOSOME	pPCL Loss %	pPCL Gain %	MM Loss %	MM Gain %	p-value
FAM46C	1p12	35	5	16	0	0.03
NRAS	1p13.2	35	0	16	0	0.03
RPL5	1p22.1	35	5	20	0	0.1
FUBP1	1p31.1	35	5	12	0	0.003
CDKN2C	1p32.3	10	5	7	0	0.6
CKS1B	1q21.3	0	45	2	31	0.2
DNMT3A	2p23.3	5	5	3	3	0.7
CRBN	3p26.2	5	5	2	2	0.4
PIK3CA	3q26.32	5	10	3	4	0.3
ADCY2	5p15.3	0	5	1	1	0.2
TNFAIP8	5q23.1	15	5	2	1	0.002
TNXB	6p21.3	10	10	2	7	0.02
PRDM1	6q21	30	0	9	1	0.002
PARK2	6q26	40	0	14	0	0.002
RAPGEF5	7p15.3	5	0	2	1	0.4
KLF14	7q32.2	0	10	0	1	0.001
DOCK5	8p21.2	15	10	16	0	<0.001
MYC	8q24.2	0	25	5	5	<0.001
CDKN2A	9p21.3	5	10	3	5	0.3
RNF20	9q31.1	0	25	3	5	<0.001
TRAF2	9q34.3	0	25	3	9	0.02
RRAS2	11p15.2	0	10	1	2	0.02
CCND1	11q13.3	0	20	0	10	0.2
BIRC3	11q22.1	10	20	4	9	0.1
ATM	11q22.3	0	25	4	9	0.02
CDKN1B	12p13.1	15	15	12	0	NS
BRCA2	13q13.1	65	0	53	0	0.3
RB1	13q14.2	65	0	55	0	NS
DIS3	13q21.33	60	5	50	0	NS
FGFR3	14p16.3	0	5	11	1	NS
ABCD4	14q24.3	25	5	32	1	NS
TRAF3	14q32.32	15	5	21	2	NS
WDR72	15q21.3	10	10	0	2	0.03
BLM	15q26.1	15	10	2	4	<0.001
CYLD	16q12.1	65	0	17	1	<0.001
WWOX	16q23.1	50	10	20	1	<0.001
MAF	16q23.2	45	0	20	1	0.002
TP53	17p13.1	35	0	8	1	<0.001
NF1	17q11.2	10	0	3	0	0.09
AKAP1	17q22	0	20	1	2	<0.001
ACTG1	17q25.3	10	10	3	1	0.001

ZNF227	19q13.3	5	5	1	2	NS
ZNF426	19p13.2	0	10	1	5	NS
CHODL	21q21.1	0	15	2	1	<0.001
SON	21q22.1	0	15	2	2	<0.001
EP300	22q13.2	20	15	11	2	<0.001

Supplemental Table 5. Codons affected by nonsynonymous mutations in *KRAS* and *TP53* of pPCL patients compared to MM. No significant differences were observed.

A. *KRAS*

Codon	pPCL (n=9)	MM (n=305)
G12D	22% (2/9)	13% (40/305)
G12A	11% (1/9)	5.6% (17/305)
Q61H	11% (1/9)	23% (69/305)
K117N	11% (1/9)	2.3% (7/305)
M67I	11% (1/9)	0% (0/305)
A59E	11% (1/9)	0.32% (1/305)
L19F	11% (1/9)	0.7% (2/305)
A146V	11% (1/9)	2.3% (7/305)

B. *TP53*

Codon	pPCL (n=11)	MM (n=81)
E294	9% (1/11)	0% (0/81)
R267W	9% (1/11)	2% (2/81)
R280K	9% (1/11)	1% (1/81)
R248W	9% (1/11)	1% (1/81)
L194F	9% (1/11)	0% (0/81)
G245S	9% (1/11)	0% (0/81)
G279E	9% (1/11)	0% (0/81)
V272M	9% (1/11)	0% (0/81)
L344P	9% (1/11)	0% (0/81)
Y234H	9% (1/11)	1% (1/81)
C277	9% (1/11)	0% (0/81)

Supplemental Table 6. A total of 126 gene probes were found differentially expressed with at least a 2 fold change between newly diagnosed pPCL and non-pPCL MM (FDR<0.01 and P<0.05)

24 significantly up-regulated genes in pPCL are listed below:

probe	gene_symbol	chrom_loc	meanMM	meanpPCL	foldchg
200916_at	TAGLN2	chr1q23.2	9.86	12.49	3.99
210978_s_at	TAGLN2	chr1q23.2	10.12	12.18	3.26
232231_at	RUNX2	chr6p21.1	8.34	10.06	3.20
210916_s_at	CD44	chr11p13	9.14	10.54	2.75
227212_s_at	PHF19	chr9q33.2	8.38	10.07	2.74
209118_s_at	TUBA1A	chr12q13.12	8.34	10.38	2.69
226961_at	PRR15	chr7p14.3	9.15	10.17	2.69
1554600_s_at	LMNA	chr1q22	8.78	10.43	2.56
200872_at	S100A10	chr1q21.3	11.09	12.71	2.47
211986_at	AHNAK	chr11q12.3	10.94	12.63	2.46
212014_x_at	CD44	chr11p13	10.26	11.47	2.34
1557905_s_at	CD44	chr11p13	10.10	11.20	2.34
201012_at	ANXA1	chr9q21.13	8.36	9.59	2.23
209835_x_at	CD44	chr11p13	10.53	11.68	2.23
227211_at	PHF19	chr9q33.2	8.13	9.81	2.18
217763_s_at	RAB31	chr18p11.22	7.83	9.18	2.16
204490_s_at	CD44	chr11p13	10.07	11.15	2.15
206632_s_at	APOBEC3B	chr22q13.1	9.58	11.04	2.12
217523_at	CD44	chr11p13	7.54	8.61	2.09
208622_s_at	EZR	chr6q25.3	10.99	12.13	2.08
209083_at	CORO1A	chr16p11.2	11.19	12.52	2.03
240983_s_at	CARS	chr11p15.4	9.22	10.12	2.03
208621_s_at	EZR	chr6q25.3	10.44	11.51	2.03
200859_x_at	FLNA	chrXq28	9.68	11.08	2.01

102 significantly down-regulated genes in pPCL are listed below:

probe	gene_symbol	chrom_loc	meanMM	meanpPCL	foldchg
214146_s_at	PPBP	chr4q13.3	9.20	6.36	6.35
215049_x_at	CD163	chr12p13.31	8.05	5.54	5.12
209116_x_at	HBB	chr11p15.4	11.39	8.98	4.76
203868_s_at	VCAM1	chr1p21.2	9.28	6.90	4.68
203645_s_at	CD163	chr12p13.31	8.59	6.34	4.27
211696_x_at	HBB	chr11p15.4	12.13	9.97	4.09

217232_x_at	HBB	chr11p15.4	11.83	9.79	3.78
209687_at	CXCL12	chr10q11.21	8.66	7.24	3.55
203698_s_at	FRZB	chr2q32.1	11.27	8.49	3.45
225353_s_at	C1QC	chr1p36.12	8.55	6.76	3.39
217378_x_at	AC016745.2 /// OTTHUMG00000153338	chr2q13	12.35	10.31	3.29
201427_s_at	SEPP1	chr5p12	11.78	10.08	3.10
213975_s_at	LYZ	chr8p11.21	8.96	7.39	3.06
227265_at	FGL2	chr7q11.23	8.33	7.36	3.02
216834_at	RGS1	chr1q31.2	11.38	10.09	2.99
234415_x_at	—	—	9.94	7.96	2.91
203697_at	FRZB	chr2q32.1	11.92	9.63	2.87
223343_at	MS4A7	chr11q12.2	8.11	6.64	2.76
229819_at	A1BG	chr19q13.43	7.75	6.09	2.76
206111_at	RNASE2	chr14q11.2	8.93	7.42	2.75
211633_x_at	—	chr14q32.33	10.08	8.15	2.74
225207_at	PDK4	chr7q21.3	8.19	7.01	2.69
211474_s_at	SERPINB6	chr1p34.1	10.37	8.99	2.67
226818_at	MPEG1	chr11q12.1	9.26	7.70	2.67
224724_at	SULF2	chr20q13.12	11.89	8.71	2.66
203535_at	S100A9	chr1q21.3	8.15	6.87	2.65
217480_x_at	AC127391.1 /// AC128677.4 /// IGKV1OR-2 /// IGKV1OR10-1 /// IGKV1OR2-118 /// OTTHUMG00000155081 /// OTTHUMG00000155090	chr10q11.21	12.63	10.94	2.64
214719_at	SLC46A3	chr13q12.3	8.86	7.35	2.61
205033_s_at	DEFA1 /// DEFA1B /// DEFA3	chr8p23.1	9.19	7.87	2.60
202252_at	RAB13	chr1q21.3	10.27	8.76	2.59
202988_s_at	RGS1	chr1q31.2	10.01	8.93	2.59
208983_s_at	PECAM1	—	12.38	10.55	2.57
217179_x_at	—	chr22q11.22	10.92	9.31	2.53
219525_at	SLC47A1	chr17p11.2	9.49	7.29	2.52
205863_at	S100A12	chr1q21.3	8.27	7.06	2.51
226841_at	MPEG1	chr11q12.1	7.98	6.65	2.50
202768_at	FOSB	chr19q13.32	11.17	9.95	2.48
237561_x_at	—	chr19p13.12	8.87	6.94	2.48
202917_s_at	S100A8	chr1q21.3	10.85	9.66	2.44
208146_s_at	CPVL	chr7p14.3	8.67	7.06	2.44
209771_x_at	CD24	chrYq11.222	8.04	6.87	2.43

233555_s_at	SULF2	chr20q13.12	10.58	7.56	2.43
203665_at	HMOX1	chr22q12.3	9.48	8.29	2.41
235666_at	ITGA8	chr10p13	11.48	10.01	2.36
211990_at	HLA-DPA1	chr6p21.32	10.68	9.24	2.36
204670_x_at	HLA-DRB1 /// HLA-DRB4 /// LOC100507709 /// LOC100507714	chr6p21.32	9.83	8.59	2.32
206834_at	HBD	chr11p15.4	10.05	8.45	2.32
223044_at	SLC40A1	chr2q32.2	11.00	10.14	2.31
203381_s_at	APOE	chr19q13.32	8.69	7.42	2.30
232745_x_at	SPEF2	chr5p13.2	7.79	6.22	2.30
228592_at	MS4A1	chr11q12.2	8.71	8.00	2.29
209035_at	MDK	chr11p11.2	9.31	8.33	2.28
206460_at	AJAP1	chr1p36.32	7.80	5.71	2.28
210517_s_at	AKAP12	chr6q25.1	8.29	6.56	2.26
1556499_s_at	COL1A1	chr17q21.33	7.74	6.26	2.25
241834_at	IPW /// LOC100506948 /// SNORD107 /// SNORD115-13 /// SNORD115-26 /// SNORD115-7 /// SNORD116-28	chr15q11.2	6.27	5.01	2.24
225102_at	MGLL	chr3q21.3	8.92	8.08	2.24
208981_at	PECAM1	chr17q23.3	13.09	11.61	2.23
208982_at	PECAM1	chr17q23.3	13.49	12.02	2.23
226743_at	SLFN11	chr17q12	10.25	8.80	2.23
206207_at	CLC	chr19q13.2	8.65	7.57	2.21
229800_at	DCLK1	chr13q13.3	7.30	5.85	2.21
219888_at	SPAG4	chr20q11.22	12.18	10.80	2.19
201278_at	DAB2	chr5p13.1	8.08	6.76	2.19
201137_s_at	HLA-DPB1	chr6p21.32	9.15	7.97	2.19
202388_at	RGS2	chr1q31.2	12.13	10.98	2.17
244419_at	FRZB	chr2q32.1	8.12	6.46	2.17
222943_at	GBA3	chr4p15.2	9.69	7.69	2.17
228375_at	IGSF11	chr3q13.32	8.42	6.89	2.16
210744_s_at	IL5RA	chr3p26.2	9.93	7.67	2.16
209312_x_at	HLA-DRB1 /// HLA-DRB4 /// HLA-DRB5 /// LOC100507709 /// LOC100507714	chr6p21.32	10.17	8.98	2.15
204834_at	FGL2	chr7q11.23	9.02	8.37	2.15
225214_at	LOC100129034	chr9q33.3	9.17	7.82	2.15
214265_at	ITGA8	chr10p13	10.72	9.36	2.14
210314_x_at	TNFSF13	chr17p13.1	8.45	7.49	2.13

206121_at	AMPD1	chr1p13.2	11.87	10.48	2.12
220330_s_at	SAMSN1	chr21q11.2	10.52	9.59	2.12
226809_at	LOC100216479 /// LOC100996884	chr2q21.1	7.44	6.76	2.11
222450_at	PMEPA1	chr20q13.31	10.26	9.10	2.11
235400_at	FCRLA	chr1q23.3	11.51	10.70	2.11
230951_at	EPB41L5	chr2q14.2	6.38	5.38	2.10
208306_x_at	HLA-DRB1 /// LOC100507709 /// LOC100507714	chr6p21.32	9.79	8.71	2.10
206150_at	CD27	chr12p13.31	11.09	9.66	2.10
202973_x_at	FAM13A	chr4q22.1	11.56	10.22	2.08
228153_at	RNF144B	chr6p22.3	9.56	8.26	2.08
200665_s_at	SPARC	chr5q33.1	9.02	7.79	2.08
1558397_at	PECAM1	chr17q23.3	9.63	8.23	2.07
219799_s_at	DHRS9	chr2q31.1	10.47	9.15	2.07
205049_s_at	CD79A	chr19q13.2	10.66	9.22	2.07
244632_at	CNTN5	chr11q22.1	7.51	5.98	2.05
227404_s_at	EGR1	chr5q31.2	12.05	10.84	2.05
220059_at	STAP1	chr4q13.2	11.24	9.68	2.03
202437_s_at	CYP1B1	chr2p22.2	6.40	5.33	2.03
204058_at	ME1	chr6q14.2	7.35	6.23	2.03
226051_at	SELM	chr22q12.2	12.28	11.11	2.02
241617_x_at	—	—	8.19	7.04	2.02
209189_at	FOS	chr14q24.3	12.91	11.98	2.02
201656_at	ITGA6	chr2q31.1	11.33	9.88	2.01
202648_at	TCF3	chr20p11.23	7.86	6.77	2.01
224009_x_at	DHRS9	chr2q31.1	10.32	9.01	2.01
227834_at	TXLNB	chr6q24.1	8.11	7.07	2.00
203988_s_at	FUT8	chr14q23.3	10.93	9.23	2.00

References

1. Mikulasova A, Wardell CP, Murison A, et al. The spectrum of somatic mutations in monoclonal gammopathy of undetermined significance indicates a less complex genomic landscape than that in multiple myeloma. *Haematologica.* 2017;102(9):1617-1625.
2. Walker BA, Mavrommatis K, Wardell CP, et al. Identification of novel mutational drivers reveals oncogene dependencies in multiple myeloma. *Blood.* 2018;132(6):587-597.
3. Walker BA, Mavrommatis K, Wardell CP, et al. A high-risk, Double-Hit, group of newly diagnosed myeloma identified by genomic analysis. *Leukemia.* 2019;33(1):159-170.
